# Supplementary material for: Association between an Increased Serum CCL5 Level and Pathophysiology of Degenerative Joint Disease in the Temporomandibular Joint in Females
Source: Int J Mol Sci. 2023 Feb 1;24(3):2775. doi: 10.3390/ijms24032775 (PMC9917489; doi:10.3390/ijms24032775)

Supplementary Figure S1

Age distribution of all DJD-TMJ and control subjects.

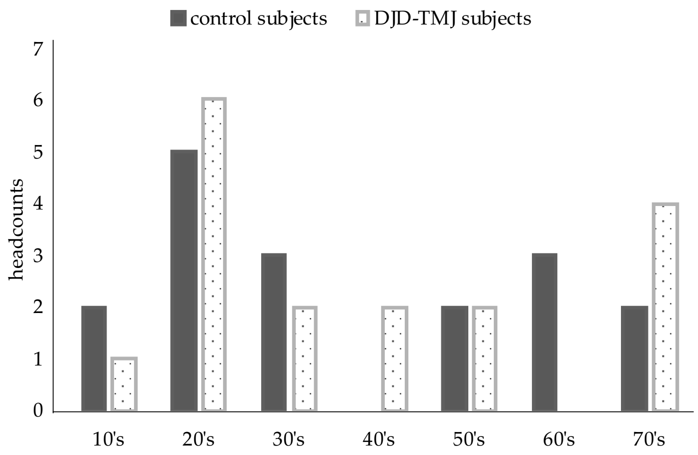

Supplementary Figure S2

Frontal view of panoramic radiograph of another representative DJD-TMJ patient than those shown in Figure 8. The letter "R" indicates right side. The bilateral resorptions of mandibular heads are indicated by arrows. Note that the right mandibular head is more severely damaged.

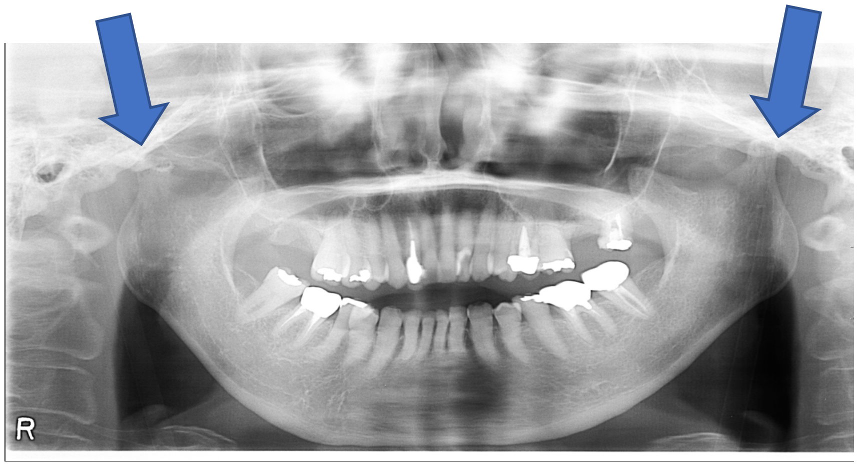

Supplement: Supplementary file 1 [file ijms-24-02775-s001.zip › ijms-2176829-supplementary.pdf]
